# Comparative analysis of chloroplast genomes and transcriptomics reveals the adaptation of *Glycyrrhiza* to salt stress

**DOI:** 10.1080/15592324.2025.2584568

**Published:** 2025-11-13

**Authors:** Mingxiang Huang, Tianxiang Zhang, Yuansheng Duan, Guifeng Zhang, Wei Hong, Yongjun Shu

**Affiliations:** a College of Traditional Chinese Medicine, Zhaoqing Medical College, Zhaoqing, Guangdong, P. R. China; b College of Life Science and Technology, Harbin Normal University, Harbin, P. R. China

**Keywords:** *Glycyrrhiza*, chloroplast genome, genetic diversity, salt stress

## Abstract

*Glycyrrhiza* is a perennial leguminous plant with salt tolerance, and its roots and rhizomes possess extremely significant medicinal value. The proper salinity can facilitate the growth of *Glycyrrhiza* and increase its content of medicinal ingredients. However, excessive salinity can negatively affect growth and medicinal component contents. The salt tolerance mechanism has not yet been fully elucidated, especially the information of chloroplast genome is in short supply. Present research investigated the genetic diversity of *Glycyrrhiza* by conducting comparative genomic, adaptive evolutionary, haplotype, population structure, and phylogenetic analyses of the chloroplast genes. Transcriptome analysis revealed that the chloroplast genes of different *Glycyrrhiza* varieties respond to salt stress at different stages and that these responsive genes are associated predominantly with the photosynthetic structure and regulation of protein synthesis. The editing efficiency of the psbA and rrn23 genes increases significantly under salt stress, potentially contributing to plant adaptation. In summary, analysis of the chloroplast genome provided valuable insights into the genetic diversity and phylogenetic relationships of *Glycyrrhiza*. Furthermore, transcriptomic data supplemented existing knowledge on chloroplast-mediated salt tolerance mechanisms, offering a foundation for future investigations into the adaptive strategies of *Glycyrrhiza* under saline conditions.

## Introduction

*Glycyrrhiza*, a perennial legume with significant medicinal value, is widely distributed in northwestern China. The dried roots and rhizomes of *Glycyrrhiza uralensis*, *Glycyrrhiza glabra*, and *Glycyrrhiza inflata* are often used as medicines.^1^*Glycyrrhiza* has a wide range of pharmacological effects, including anti-inflammatory, antibacterial, antiviral, antitumor, and multiple organ protective effects.^2^^,^^3^ It also demonstrates considerable potential for applications in both medical and animal feed fields.^4^^,^^5^ In addition to their pharmacological properties, *Glycyrrhiza* species are notable for their ability to survive in extreme environments, particularly under conditions of high salinity, drought, and intense light. This unique ecological adaptability makes them valuable model resources for studying plant salt tolerance mechanisms.^6^

In recent years, soil salinization has become increasingly severe, posing a significant threat to the natural distribution and sustainable utilization of plant resources. In China, *Glycyrrhiza* species are distributed primarily in saline–alkaline desert grasslands in arid and semiarid regions of the northwest.^7^ Although these species exhibit a certain degree of salt tolerance, for example, *Glycyrrhiza inflata* and *Glycyrrhiza uralensis* can thrive in saline‒alkaline soils. Notably, over the past two decades, the synergistic effects of overexploitation and environmental degradation have led to a marked decline in both the population density and biomass of *Glycyrrhiza*.^8^ Therefore, identifying genes associated with salt tolerance in *Glycyrrhiza* is crucial to ensure the sustainable utilization of this resource in salinized environments. Current research has made significant progress in elucidating the molecular mechanisms and physiological responses related to salt tolerance in Glycyrrhiza.^8^^-^^10^ Dong et al. systematically investigated the regulatory effects of exogenous glycine betaine (GB) on the physiological characteristics of *Glycyrrhiza* seedlings under salt stress and revealed that GB treatment significantly enhances the antioxidant capacity and osmotic regulation of these seedlings.^10^ Using high-throughput transcriptome sequencing technology, Jia et al. identified key salt tolerance-related genes, such as SAPK2, LAC3, and AHK2, which are involved in regulating ion absorption and root growth and development.^8^ However, research on the chloroplast genome of *Glycyrrhiza* remains significantly limited, greatly restricting a comprehensive understanding of salt tolerance mechanisms at the organelle level.

Chloroplasts are semiautonomous organelles that evolved from ancestral cyanobacteria through endosymbiosis, featuring a double-membrane structure with an internal thylakoid membrane system and stroma.^11^ Chloroplasts are not only the primary site for photosynthesis and Calvin cycle metabolism but also central hubs for plant stress responses.^12,^^13^ Among various abiotic stresses, salt stress significantly inhibits plant growth and development by disrupting ion homeostasis.^14^ The chloroplast genome, which is typically 120−160 kb in size and encodes approximately 100−130 genes, is highly sensitive to environmental stresses.^15^ Under salt stress, the electron transport chain becomes compromised, leading to increased accumulation of reactive oxygen species (ROS). When ROS levels exceed the scavenging capacity of antioxidant systems, they trigger cascading effects, including protein denaturation and DNA damage, ultimately impairing chloroplast integrity and the photosynthetic machinery.^13,^^14^ Moreover, the chloroplast genome has become an important tool for studying plant phylogenetics and molecular evolution due to its structural conservation, maternal inheritance, low evolutionary rate and limited recombination. ^16,^^17^

Recent breakthroughs in population chloroplast genomics include phylogenetic and population genetic analyses of 1,579 Brassica napus that elucidated multiple origin patterns of its chloroplast genome.^18^ However, systematic investigations of the population genetic structure, haplotype diversity, and adaptive evolution characteristics of the *Glycyrrhiza* chloroplast genome are lacking. Research has focused primarily on the genome assembly and annotation of different *Glycyrrhiza* varieties. For instance, Wu et al. conducted comparative and phylogenomic analyses of six *Glycyrrhiza* chloroplast genomes, revealing features such as the loss of inverted repeats, and performed preliminary analyses of selection pressure on genes.^19^ Jiang et al. characterized the transcriptome and complete chloroplast genome of *Glycyrrhiza inflata* and conducted comparative analyses with those of *Glycyrrhiza uralensis* and *Glycyrrhiza glabra*.^20^ Therefore, systematic studies of the chloroplast genomes of *Glycyrrhiza* varieties are needed to elucidate their population genetic structure and adaptive evolutionary patterns, which are crucial for understanding the genetic diversity and salt tolerance mechanisms of *Glycyrrhiza* varieties.

In this study, the chloroplast genomes of six different *Glycyrrhiza* varieties were collected and analyzed using comparative genomics to investigate their structural characteristics. In addition, 60 publicly available *Glycyrrhiza* chloroplast genome datasets were integrated to explore patterns of genomic variation. Based on haplotype analysis, phylogenetic, and population genetic structure evaluation, the genetic diversity of *Glycyrrhiza* chloroplast genomes was comprehensively characterized. Furthermore, transcriptome sequencing technology was employed to elucidate the regulatory patterns of chloroplast gene expression under salt stress conditions. Based on these datasets, this study aims to explore the phylogenetic history and evolutionary history of *Glycyrrhiza* and to identify key functional genes under positive selection along with their adaptive evolutionary mechanisms. These findings provide a foundation for a deeper understanding of the genetic diversity and phylogenetic evolution of *Glycyrrhiza*.

## Materials and methods

### Divergence analysis of the whole chloroplast genes of the genus *Glycyrrhiza*

The chloroplast genomes of six *Glycyrrhiza* varieties (MH321931, MN562092, KY038482, KU862308, PP119340, and PP119343) were collected from the NCBI database (https://www.ncbi.nlm.nih.gov/) (accessed on 5 August 2024). The mVISTA online software (https://genome.lbl.gov/vista/index.shtml) (accessed on 5 September 2024)^21^ was utilized to conduct a sequence comparative analysis, with MN199032 as the reference chloroplast genome. In the shuffle-LAGAN model, the six varieties were compared. The minimum and maximum values of the y-axis were set from 50% to 100%, and the sliding window size was set to 100 bp.

### SNP analysis of chloroplast genes in *Glycyrrhiza uralensis*

The original sequence data of 60 *Glycyrrhiza* DNA resequencing datasets were collected from the SRA database (https://www.ncbi.nlm.nih.gov/sra) (PRJNA730103; accessed on 17 August 2024). The fastq-dump tool from the SRA Toolkit (https://ftp-trace.ncbi.nlm.nih.gov/sra/sdk/2.9.6/) (accessed on 27 August 2024) was used to extract the fastq files of the DNA sequencing data. Quality control and adapter trimming of the raw reads were performed using Trimmomatic (version 0.3.2).^22^ The filtered reads from the 60 resequenced datasets were independently mapped to the MN199032 chloroplast genome using Burrows-Wheeler Aligner software (version 0.7.17).^23^ The resulting alignment files in SAM format were converted into BAM (the binary version of SAM) format using (version 0.7.17).^24^ The addOrReplaceReadGroups tool of GATK (version 4.4.0.0)^25^ was used to add read groups, and then the MarkDuplicates tool was employed to remove duplicates. Joint genotyping of the gVCF files was conducted using the HaplotypeCaller tool, after which the GenotypeGVCFs tool was used to convert the gVCF files into VCF format. SNPs and INDELs were subsequently filtered using the SelectVariants function. The alignment of the *Glycyrrhiza* chloroplast genomes was carried out with MAFFT (version 7.526)^26^ using default parameters. Nucleotide diversity (Pi) values were calculated using DnaSP 6^27^ with a step size of 200 bp and a window length of 1,000 bp to assess sequence variation. Based on the sequence alignment, Ka and Ks substitution rates, along with Ka/Ks ratios for all chloroplast genes, were estimated using KaKs_Calculator 3.0.^28^

### Haplotype analysis

Using MN199032 as the reference, the chloroplast genomes of *Glycyrrhiza* were constructed with BCFtools (version 1.19.2) based on the variant sites identified in 60 *Glycyrrhiza uralensis* samples. Multiple sequence alignment of *Glycyrrhiza* nucleotide sequences was performed using MAFFT (version 7.526).^26^ Haplotype numbers were determined using DnaSP 6 software.^27^ Furthermore, haplotype diversity was assessed, and a haplotype network was constructed using POPART (version 1.7).^29^

### Population structure analysis

SNP filtering was performed using VCFtools (version 0.1.16)^30^ with the following parameters: missing rate <  50% and minor allele frequency > 0.05. VCF-formatted files were converted to PHYLIP format using PLINK (version 7.505).^31^ Multiple sequence alignment was conducted on the PHYLIP-formatted files using MAFFT software (version 7.526).^26^ In IQ-TREE2 (version 2.0)^32^, the bootstrap value was set to 1000 to construct the maximum likelihood (ML) phylogenetic tree. The resulting tree file was visualized using FigTree (version 1.4.4).^33^ Population structure analysis of SNPs from 60 *Glycyrrhiza* samples was conducted using ADMIXTURE (version 1.3.0), with the number of genetic clusters (K) set to range from 2 to 10. Visualization for K = 2, K = 3, and K = 4 was performed in R.^34^

### Analysis of RNA-Seq and RNA editing efficiency

Twelve RNA-seq datasets were obtained from the SRA database (https://www.ncbi.nlm.nih.gov/sra) (PRJNA977447; accessed on 17 August 2024). Clean reads from these samples were mapped to the MN199032 chloroplast genome using Salmon (version 2.0.1).^35^ Transcript abundance was quantified in transcripts per million (TPM) using default parameters and visualized with R packages. For the 12 samples, raw sequencing reads were mapped to the MN199032 chloroplast genome via the Burrows–Wheeler Aligner (version 0.7.17).^23^ High-quality SNPs were obtained through standard GATK (version 4.4.0.0) pipeline filtering. RNA editing sites were detected from SNPs using the REDO software (version 1.0), and the results were visualized using R packages.^36^

## Results

### Comparative analysis of chloroplast genes in the genus *Glycyrrhiza*

**Figure 1.**
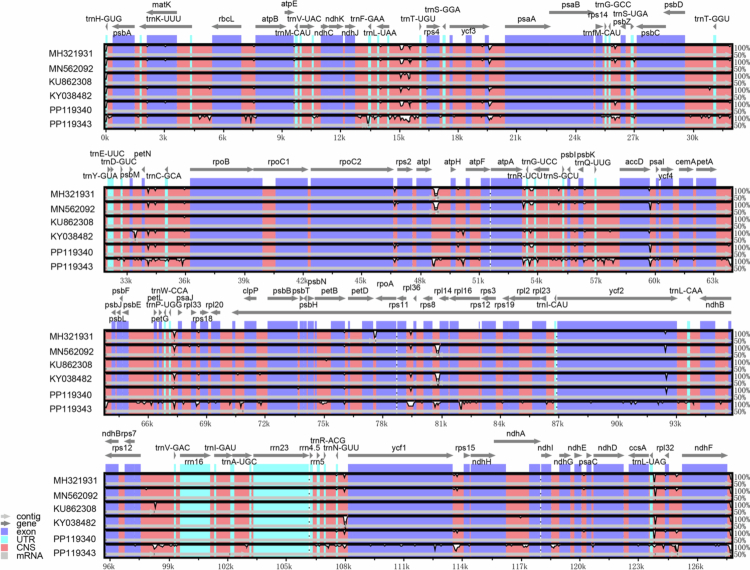
Comparative analysis of chloroplast genes in six *Glycyrrhiza* varieties. Note: With the chloroplast genome sequence of MN199032 (*Glycyrrhiza uralensis*) as a reference, the complete chloroplast genome sequences of the *Glycyrrhiza* varieties were examined. The transcriptional orientation of genes is depicted by gray arrows and bold black lines. Exonic regions are marked with purple rectangles, untranslated regions (UTRs) with light blue bars, and conserved noncoding sequences (CNS) with red bars. mRNA-corresponding segments are shown as gray bars, while white areas display sequence variations among all compared chloroplast genomes. The x-axis indicates nucleotide positions along the chloroplast genome, and the y-axis represents the sequence homology percentage, scaled from 50% to 100%.

To better compare the variation in chloroplast genomes among different *Glycyrrhiza* varieties, we conducted a whole-genome comparative analysis using mVISTA software^21^ (https://genome.lbl.gov/vista/index.shtml) (accessed on 5 September 2024). The chloroplast genome sequence MN199032 was used as the reference, alongside six chloroplast genomes of *Glycyrrhiza* varieties, including MH321931, MN562092, KY038482, KU862308, PP119340, and PP119343 (Figure 1, Table S1). Sequence variation in non-coding regions was primarily concentrated in the 15–27, 30–36, 46–60, 66–69, 79–81, 108, and 124 kb regions, while coding regions of genes such as rps4, accD, petB, ycf1, ycf2, and ndhF also presented detectable sequence differences. Overall, the high conservation of coding regions alongside variable noncoding regions suggests that such genomic structural differences may influence gene expression and photosynthetic efficiency, potentially contributing to the adaptation of *Glycyrrhiza* to saline environments.

### Adaptive evolution of the chloroplast genomes in *Glycyrrhiza uralensis*

**Figure 2.**
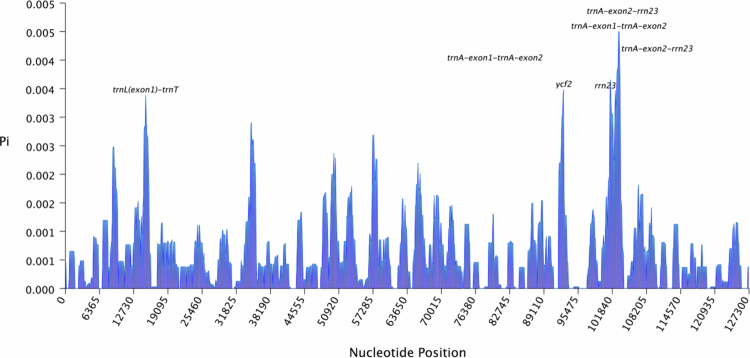
Nucleotide diversity (Pi) of the chloroplast genomes of 60 *Glycyrrhiza uralensis* accessions. Note: The horizontal axis represents the nucleotide position, and the vertical axis indicates the nucleotide diversity at each site. The analysis was performed with a 200 bp step size and a 1000 bp window length.

**Figure 3.**
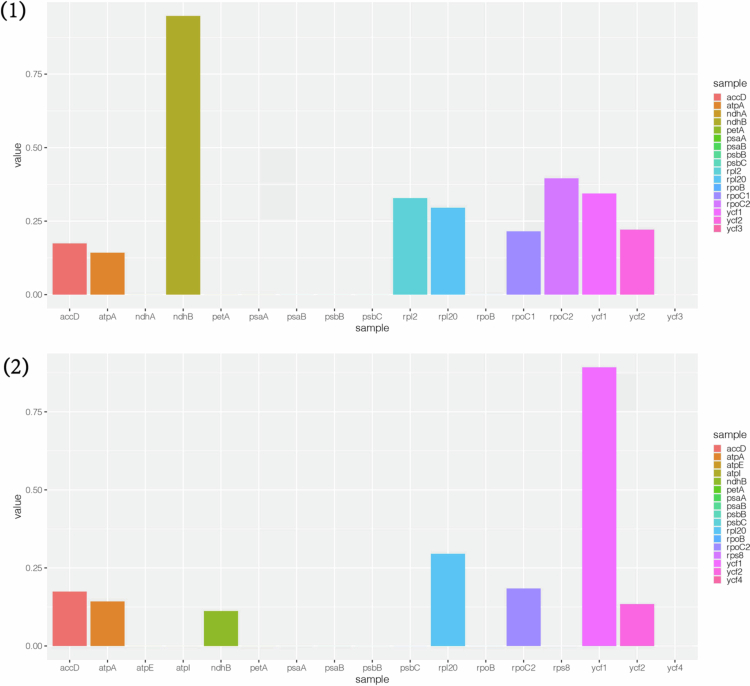
(1) Evolutionary values of the Ka/Ks ratio in 60 *Glycyrrhiza uralensis*. (2) Evolutionary values of the Ka/Ks ratios of the 6 *Glycyrrhiza* varieties. Note: The Ka/Ks ratios for all genes of 60 *Glycyrrhiza uralensis* and 6 *Glycyrrhiza* varieties were less than 1.

Resequencing data from 60 genomes of *Glycyrrhiza uralensis* collected from the central, eastern, and southern regions of Xinjiang Province, China,^6^ were analyzed to calculate the nucleotide diversity (Pi) values of these chloroplast genomes (Table S2). The results revealed that intergenic regions presented relatively higher nucleotide diversity compared to coding regions did. Significantly higher Pi values (>0.005) were particularly observed in the trnA-exon2-rrn23 and trnA-exon1-trnA-exon2 regions (Figure 2). To investigate the adaptive evolution of *Glycyrrhiza*, the Ka/Ks ratios of protein-coding genes were analyzed in 60 *Glycyrrhiza uralensis* chloroplast genomes, along with six *Glycyrrhiza* varieties involved in the chloroplast genome comparison (Figure 3). The visualized results revealed that the Ka/Ks ratios for all the genes were less than 1, indicating the dominance of purifying selection. These findings suggest a high degree of conservation in the evolutionary processes of *Glycyrrhiza* chloroplast genes. The variability of non-coding regions, in conjunction with the high conservation of functional genes, constitutes a characteristic feature of the *Glycyrrhiza* chloroplast genome, which may enhance adaptation to saline environments by modulating photosynthesis and gene expression.

### Haplotype analysis of chloroplast genes

**Figure 4.**
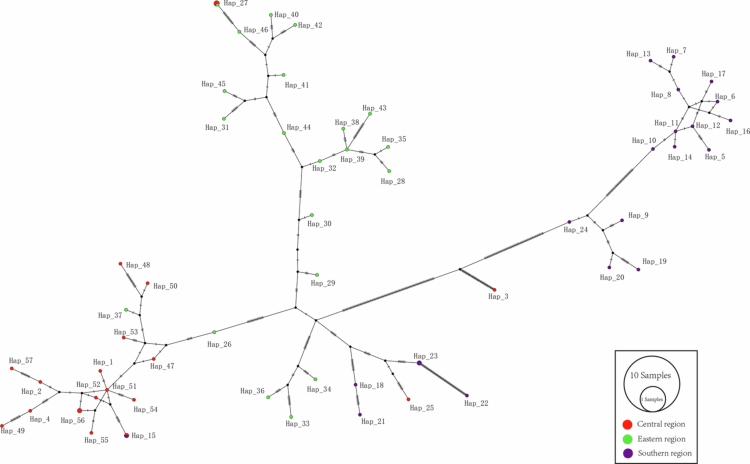
The haplotype network was constructed based on the variant sites of 60 *Glycyrrhiza* *uralensis* genomes. Note: The colors used in the figure are as follows: red represents varieties from the central region, green represents varieties from the eastern region, and purple represents varieties from the southern region. In the haplotype network structure, each node represents a distinct haplotype, with its size proportional to the number of individuals sharing that haplotype. The connecting lines between nodes indicate relationships between haplotypes. The color scheme of the nodes in the network is consistent with that in the phylogenetic tree, ensuring visual coherence.

The chloroplast genomes of 60 *Glycyrrhiza uralensis* plants were used for haplotype analysis, resulting in the identification of 57 haplotypes (Figure 4; Table S3). Based on geographical distribution, the *Glycyrrhiza uralensis* population was divided into three groups. The classification of haplotypes largely aligned with the geographical distribution. The central region comprised 18 haplotypes, the eastern region included 21 haplotypes, and the southern region contained 20 haplotypes. However, several haplotypes, such as Hap_27 and Hap_15, were shared across regions, indicating potential gene flow and hybridization among populations from different regions. The haplotype analysis revealed that Hap_37, typically associated with the central region, was specific to the eastern region, whereas Hap_3 and Hap_25, assigned to the southern region, were also found in the central region. The diversity and interregional distribution patterns of the chloroplast haplotypes suggest that gene flow and local adaptation may have jointly shaped the population structure of *Glycyrrhiza uralensis* and that such genetic diversity may enhance its adaptive capacity under saline stress conditions.

### Analysis of population genetic structure

**Figure 5.**
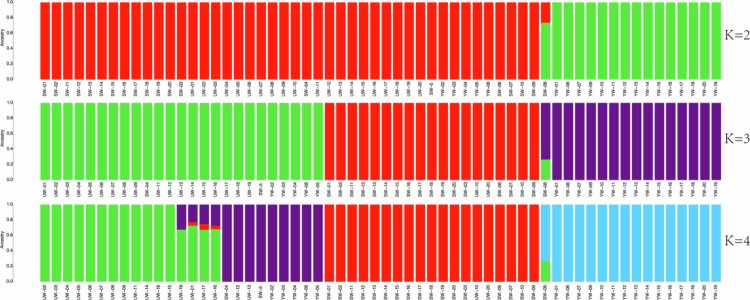
Population structure analysis of the chloroplast genomes of 60 *Glycyrrhiza uralensis* based on SNPs. Note: The colors used in the figure are as follows: red represents varieties from the central region, green represents varieties from the eastern region, and purple represents varieties from the southern region. In fact, UW represents varieties from the eastern regions, SW represents varieties from the central region, and YW represents varieties from the southern region.

Population structure refers to the varying degrees of genetic relatedness among subgroups within a species [36]. Cross-validation (CV) error values were calculated for K values between 2 and 10 during the admixture-based population structure analysis of *Glycyrrhiza uralensis*. The optimal number of ancestral components was determined by identifying the K value with the minimum CV error. The figure illustrates the genetic composition of the population for K values ranging from 2 to 4 (Figure 5). The *Glycyrrhiza uralensis* population was assigned to three major clades, with the minimum CV error observed at K = 3. Notably, the eastern group includes two varieties from the central region, and the central group includes two varieties from the eastern region. All southern varieties are grouped together, which is largely consistent with the results from the haplotype analysis and phylogenetic tree construction. This interpopulation genetic admixture may reflect historical germplasm exchange and ecological migration processes, which have contributed to the accumulation of important genetic foundations for the survival and adaptation of *Glycyrrhiza uralensis* in saline environments.

### Phylogenetic tree based on SNPs

**Figure 6.**
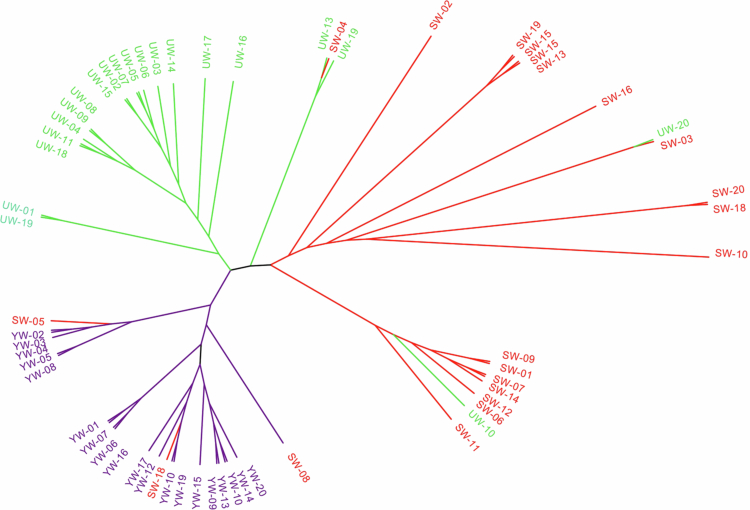
The phylogenetic tree was constructed based on the SNPs of the genomes of 60 *Glycyrrhiza uralensis* from Xinjiang Province, China. Note: The central varieties are represented by red, the eastern varieties are represented by green, and the southern varieties are represented by purple.

The 60 chloroplast genomes of *Glycyrrhiza uralensis* were compared with the chloroplast genome of MN199032, resulting in the identification of 422 SNPs and the construction of a maximum likelihood (ML) phylogenetic tree based on the constant sites (Figure 6). The phylogenetic results based on SNP sites are basically consistent with the results from haplotype analysis and population origin. The phylogenetic tree is divided into three primary branches, representing the central, eastern, and southern regions. UW−20 and UW−10 from the eastern region were assigned to the central genetic clade, while SW−04 from the central region clustered with the eastern lineage. Notably, the central accessions SW−05, SW−08, and SW−18 were grouped within the southern clade. The distribution of these SNPs is highly consistent with the previous haplotype and population structure analyses. Variation in the chloroplast genome not only maintains regional genetic differentiation but also promotes overall genetic diversity through gene flow, thereby enhancing the adaptation of *Glycyrrhiza* to saline environments.

### Responses of chloroplast genes to salt stress in *Glycyrrhiza*

**Figure 7.**
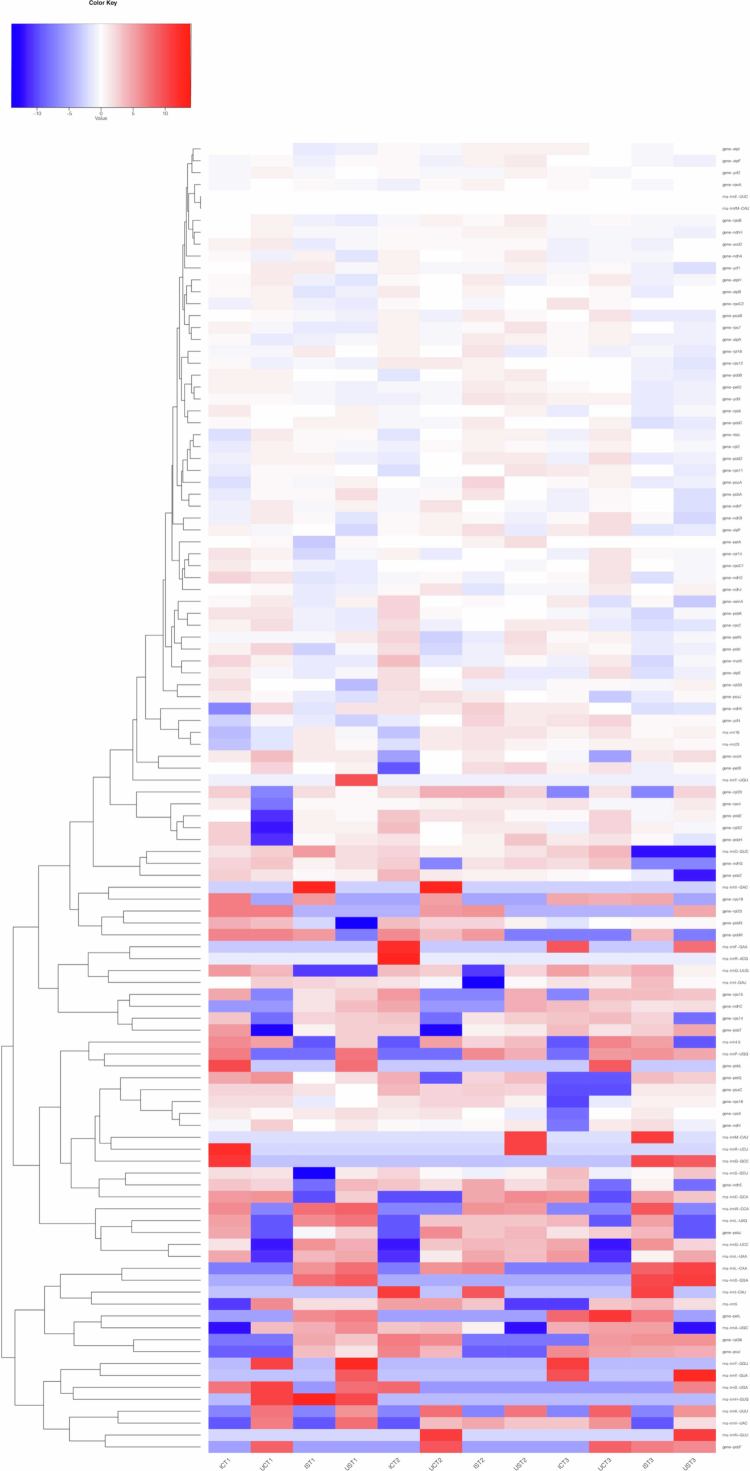
Heatmap of chloroplast gene expression in *Glycyrrhiza inflata* and *Glycyrrhiza uralensis* under salt stress. Note: IST1–3 represent *Glycyrrhiza inflata* subjected to salt stress for 0.5 d, 15 d, and 30 d, respectively, while ICT1–3 denote *Glycyrrhiza inflata* under control conditions for the same time points. Similarly, UST1–3 correspond to *Glycyrrhiza uralensis* exposed to salt stress for 0.5 d, 15 d, and 30 d, whereas UCT1–3 indicate *Glycyrrhiza uralensis* maintained under control conditions at the same time intervals.

Chloroplasts are sites where photosynthesis and various biochemical reactions occur and are highly sensitive to salt stress.^37^ Chloroplasts have evolved intricate adaptive mechanisms to acclimate to salt stress. Twelve transcriptome datasets of the chloroplast genomes were analyzed from the *Glycyrrhiza uralensis* and *Glycyrrhiza inflata* varieties (Figure 7). The genes psal, trnA-UGC, rpl36, petL, trns-GGA, trnL-CAA and ndhC in *Glycyrrhiza uralensis* and *Glycyrrhiza inflata* rapidly responded to salt stress, with significant upregulation observed at 0.5 d. The expression levels of trnN-GUU, psbF, and trnG-GCC were significantly downregulated at 0.5 d and 15 d but were upregulated at 30 d. This pattern suggests that the expression of these genes was inhibited in the early stages, but as time progressed, their expression gradually recovered and increased by 30 d, likely reflecting plant adaptation or recovery mechanisms in response to external conditions. Genes such as rnl-UAA, trnG-UCC, psbJ, and trnL-UAG were significantly upregulated in the early stages of salt stress in *Glycyrrhiza uralensis*, while in *Glycyrrhiza inflata*, these genes were upregulated only in the middle stage of salt stress.

**Figure 8.**
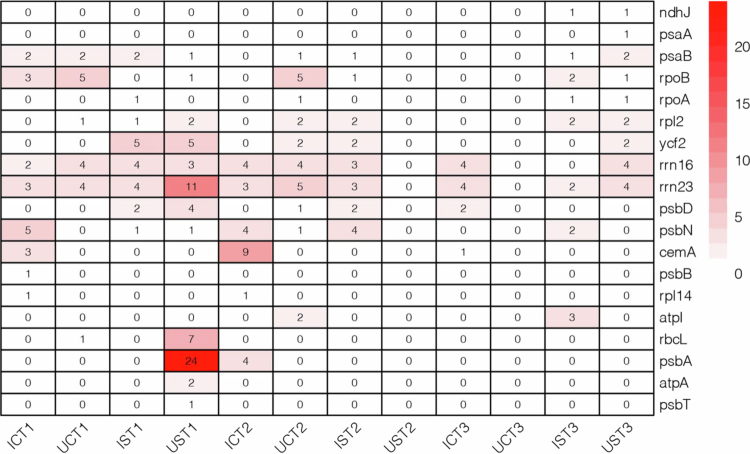
The RNA editing efficiency of chloroplast genes under salt stress was analyzed in *Glycyrrhiza inflata* and *Glycyrrhiza uralensis*. Note: The experimental groups were defined as follows: IST1−3 represent *Glycyrrhiza inflata* subjected to salt stress for 0.5 d, 15 d, and 30 d, respectively; ICT1−3 denotes *Glycyrrhiza inflata* under control conditions for the same time points. Similarly, UST1−3 correspond to *Glycyrrhiza uralensis* exposed to salt stress at 0.5 d, 15 d, and 30 d, while UCT1−3 refer to *Glycyrrhiza uralensis* maintained under control conditions for the corresponding durations.

The data from twelve transcriptomes were used to analyze RNA editing events in chloroplasts (Figure 8; Table S4). A total of 19 genes undergoing editing events were identified, with C-to-T editing being the most prevalent in chloroplast genomes. During the early stage of salt stress, 12 editing events were detected in *Glycyrrhiza uralensis*, whereas only 6 editing events were observed in the negative control group. Among the genes subjected to RNA editing, psbA exhibited the highest number of editing sites,^24^ followed by rrn23, with 11 sites. These findings suggest that RNA editing in *Glycyrrhiza uralensis* facilitates a rapid response during the early stage of salt stress, potentially contributing to the repair capacity of the photosystem, ribosome assembly and protein synthesis. Chloroplast genes in *Glycyrrhiza* exhibit time-dependent dynamic regulation, with genes initially suppressed during the early stages gradually upregulated, indicating the activation of pathways related to photosynthesis, protein synthesis, and energy metabolism in response to salt stress. Meanwhile, RNA editing events increase significantly in the early stages of salt stress, potentially facilitating photosystem repair and ribosome assembly, thereby enhancing the rapid response and adaptation of *Glycyrrhiza* under salt stress.

## Discussion

As an important tool for studying plant evolution and phylogeny, the chloroplast genome revealed a balance between conservation and variation through comparative analysis of the six *Glycyrrhiza* varieties in this study. The chloroplast genomes of all six varieties were approximately 127  kb in size, indicating a highly conserved genomic structure, which is consistent with findings from previous studies on the chloroplast genomes of various plant species.^38^ Genomic variations were primarily concentrated in non-coding regions, a pattern commonly observed in comparative analyses of plant chloroplast genomes. This study identified multiple highly variable noncoding regions in *Glycyrrhiza*, particularly in certain intronic regions that presented significant polymorphisms. Nucleic acid diversity analysis revealed seven variable regions, including five intergenic regions and two genic regions. These regions may play crucial roles in regulating the expression of neighboring genes, and their variations could serve as regulatory reservoirs, enabling plants to respond rapidly to abiotic stress through epigenetic or posttranscriptional regulatory mechanisms.^39^ Although coding regions generally remained conserved, significant interspecific divergence was observed in genes such as accD, ycf1 and ycf2. These genes may have undergone species-specific selective pressures, and their variation patterns reflect the adaptive evolution of *Glycyrrhiza* to diverse ecological environments. Variable regions of the chloroplast genome can serve as candidate markers for plant DNA barcoding, enabling rapid species identification. In Taxus, the accD gene and the rrn16–rrn23 intergenic spacer have been confirmed as effective and specific barcodes for distinguishing species.^40^ Studies have shown that the ycf2 and ycf1 genes exhibit significant variation among different species, with structural variation and even complete loss observed in Passiflora.^41^ This suggests the possible existence of alternative translation systems and motor protein complexes that compensate for the loss of these genes. The functional redundancy of the ycf1 gene allows it to accumulate a certain degree of genetic variation, fully demonstrating the plasticity of the chloroplast genome during evolution. This study further revealed the unique variation patterns of these variable genes in *Glycyrrhiza*, providing new insights into the adaptive evolution of the chloroplast genome.

Ka/Ks analysis was performed on the chloroplast genomes of 60 *Glycyrrhiza uralensis* varieties and six different *Glycyrrhiza* varieties. The results revealed that the Ka/Ks ratios of all protein-coding genes were less than 1, indicating strong purifying selection acting on the chloroplast genomes of *Glycyrrhiza*. Notably, the ndhB gene in *Glycyrrhiza uralensis* and the ycf1 gene across the six *Glycyrrhiza* varieties presented ratios close to the neutral selection threshold, suggesting that these genes may play important roles in adaptive evolution. The ndhB complex, as a key component of the chloroplast electron transport chain, plays a crucial role in cyclic electron flow, thereby regulating photosynthetic efficiency.^42^ Additionally, ndhB is closely associated with plant responses to abiotic stress. The elevated Ka/Ks ratio of ndhB may indicate that this gene is adapting to environmental pressures or has undergone functional changes driven by natural selection, providing an evolutionary basis for physiological regulation in plants.^13^ Ka/Ks analysis revealed that while the chloroplast genomes of *Glycyrrhiza* are under strong purifying selection overall, potential functional variations in the ndhB and ycf1 genes offer important insights into the adaptive evolution of *Glycyrrhiza*. These findings provide valuable clues for further exploration of the molecular evolution of key functional genes and the mechanisms underlying the adaptive evolution of *Glycyrrhiza*.

Among the 60 chloroplast genomes of *Glycyrrhiza uralensis*, a total of 57 haplotypes were identified, indicating high genetic diversity within the population. Based on haplotype network analysis, the varieties were divided into three distinct groups. These groups showed a significant correlation with geographical distribution, leading to their classification into three regions: eastern Glycyrrhiza, southern Glycyrrhiza, and central Glycyrrhiza. The phylogenetic tree constructed using SNPs from *Glycyrrhiza* was consistent with previous studies based on nuclear genomes, validating the robustness of these markers.^6^ The chloroplast genome has a conserved structure and a moderate mutation rate, making its SNP markers potentially highly versatile for closely related or widely distributed species^16,^^17^ Given that the chloroplast genome is maternally inherited, its variation patterns can trace maternal lineage history and reveal evolutionary dynamics more rapidly than nuclear genome data.

Salt stress affects plant growth and development through multiple physiological mechanisms. It can lead to structural damage in chloroplasts, dysfunction of the photosynthetic electron transport chain, disruption of cellular osmotic regulation, and disturbances in reactive oxygen species metabolism.^43^^,^^44^ Taking *Glycyrrhiza inflata* and *Glycyrrhiza uralensis* as examples, studies have shown that after 0.5 d of salt stress treatment, the expression levels of multiple genes in their chloroplast genomes, such as psaA, trnA-UGC, rpl36 and petL, are significantly upregulated, suggesting that these genes may be involved in the early response to salt stress. Research has shown that the protein encoded by the psaA gene helps ferns adapt to the new photosynthetic environment following the rise of angiosperms.^45^ Moreover, studies on rice have demonstrated that the overexpression of the rpl6 gene, which encodes a large ribosomal subunit protein, significantly enhances plant tolerance to 150–200 mM salt stress.^46^ Based on sequence and functional similarity, the homologous rpl36 gene may play a similar role in salt tolerance in *Glycyrrhiza* varieties. Additionally, genes involved in photosynthetic electron transport, such as petM and petL, also exhibit salt stress-responsive characteristics.^47^ Notably, petL is particularly significantly upregulated early in *Glycyrrhiza uralensis*, suggesting that this species may rapidly adjust the expression of electron transport chain-related genes to cope with salt stress. Interestingly, different *Glycyrrhiza* varieties exhibit temporal variations in their gene responses to salt stress. For instance, the trnL-UAA and psbJ genes were significantly upregulated in *Glycyrrhiza uralensis* as early as 0.5 d, whereas their upregulation in *Glycyrrhiza inflata* was delayed until 15 d. This difference in response speed may be one of the key factors contributing to the variation in salt tolerance between the two species. Moreover, the expression of certain genes, such as psbF, displays dynamic patterns—downregulated in the early stages (0.5 d and 15 d) but upregulated later (30 d)—reflecting the phased regulatory strategy of *Glycyrrhiza* in response to salt stress.

RNA editing serves as a crucial posttranscriptional regulatory mechanism in chloroplast genomes, which precisely modulates chloroplast development and physiological functions through specific base modifications that alter RNA coding sequences.^48^ This mechanism significantly enhances the phenotypic plasticity of plant genomes, representing a vital molecular basis for environmental adaptation.^49^ The results of the present study revealed that salt stress induced RNA editing in 19 chloroplast genes in *Glycyrrhiza uralensis*, with C-to-T being the predominant type. A twofold increase in editing sites was detected during the early stress phase (0.5 d; 12 sites in the treatment group vs. 6 in the control group), including 24 high-density editing sites in psbA and 11 editing sites in rrn23. In mature chloroplasts, the light-dependent formation of ribosome-psbA mRNA complexes not only accelerated the synthesis of the D1 protein, a key factor in photosystem II (PSII) repair, but also substantially increased global translational efficiency through systemic regulation.^50^ Transcriptome profiling further demonstrated sustained upregulation of photosystem-related psaL and psbJ genes throughout the salt stress response.

This study revealed that different *Glycyrrhiza* varieties presented significant differences in chloroplast gene expression and RNA editing patterns under salt stress. These temporal and intensity variations likely reflect the evolution of salt tolerance in each variety. Rapidly upregulated genes and early RNA editing events may provide molecular support for photosystem repair and photosynthetic homeostasis, thereby increasing the survival capacity of these species under salt stress. These findings not only reveal the functional dynamics of *Glycyrrhiza* chloroplast genomes in response to salt stress but also provide important insights into the evolutionary mechanisms underlying interspecies differences in salt tolerance.

## Conclusion

This study conducted a comparative analysis of the chloroplast genomes of different *Glycyrrhiza* varieties, with the main findings summarized as follows: (1) the chloroplast genomes are generally conserved, with seven hypervariable regions identified; (2) genes such as ndhB and ycf1 may exhibit signatures of adaptive evolution; (3) RNA editing, particularly in psbA and rrn23, increases significantly during the early stages of salt stress, facilitating photosystem repair; and (4) different *Glycyrrhiza* varieties display distinct gene expression patterns under salt stress, reflecting potential differences in salt tolerance. These results provide candidate genes and molecular markers for the molecular breeding of salt-tolerant *Glycyrrhiza* and offer a theoretical basis for the identification and improvement of related functional genes.

## Supplementary Material

Supplementary material Table S4 Statistics on RNA editing sites in *Glycyrrhiza*.

Supplementary material Table S3 Statistical analysis of haplotype numbers in sixty *Glycyrrhiza uralensi*.

Supplementary material Table S2 Information of sixty *Glycyrrhiza uralensis*.

Supplementary material Table S1 Information of seven *Glycyrrhiza* varieties.

## Data Availability

The datasets presented in this study can be found in the Supplementary Material.

## References

[1] Shang Z, Liu C, Qiao X, Ye M. Chemical analysis of the Chinese herbal medicine licorice (Gan-Cao): an update review. J Ethnopharmacol. 2022;299:115686. doi: 10.1016/j.jep.2022.115686. 36067839

[2] Hosseinzadeh H, Nassiri-Asl M. Pharmacological effects of *Glycyrrhiza* spp. and its bioactive constituents: update and review. Phytother Res. 2015;29(12):1868–1886. doi: 10.1002/ptr.5487. 26462981

[3] Yo YT, Shieh GS, Hsu KF, Wu CL, Shiau AL. Licorice and licochalcone-A induce autophagy in LNCaP prostate cancer cells by suppression of Bcl-2 expression and the mTOR pathway. J Agric Food Chem. 2009;57(18):8266–8273. doi: 10.1021/jf901054c. 19711916

[4] Liu W, Huang S, Li Y, Wu P, Wang Q, Zheng X, Zhang K. Glycyrrhizic acid from licorice down-regulates inflammatory responses via blocking MAPK and PI3K/Akt-dependent NF-κB signalling pathways in TPA-induced skin inflammation. MedChemComm. 2018;9(9):1502–1510. doi: 10.1039/C8MD00288F. 30288224 PMC6148683

[5] Dang H, Zhang T, Li Y, Zhuang L, Pu X. Population evolution, genetic diversity and structure of the medicinal legume, *Glycyrrhiza uralensis* and the effects of geographical distribution on leaves nutrient elements and photosynthesis. Front Plant Sci. 2022;12:708709. doi: 10.3389/fpls.2021.708709. 35069610 PMC8782460

[6] Luiz AC. Beyond conservation: the landscape of chloroplast genome rearrangements in angiosperms. New Phytol. 2025;247(6):2571–2580. doi: 10.1111/nph.70364. 40613318 PMC12371153

[7] Li Q, Wang X, Yan K, Liang Z, Xia P. Based on multiple environmental factors to explore the habitat distribution of Licorice (*Glycyrrhiza uralensis*) in different time and space. Biochem Syst Ecol. 2022;5:104490. doi: 10.1016/j.bse.2022.104490.

[8] Xu Y, Lu JH, Zhang JD, Liu D, Wang Y, Niu Q, Huang D. Transcriptome revealed the molecular mechanism of *Glycyrrhiza inflata* root to maintain growth and development, absorb and distribute ions under salt stress. BMC Plant Biol. 2021;21(1):599. doi: 10.1186/s12870-021-03342-6. 34915868 PMC8675533

[9] Jia T, Gu J, Ma M. La (NO3)3 substantially fortified *Glycyrrhiza uralensis*'s resilience against salt stress by interconnected pathways. BMC Plant Biol. 2024;24(1):926. doi: 10.1186/s12870-024-05644-x. 39367329 PMC11452937

[10] Dong X, Ma X, Zhao Z, Ma M. Exogenous betaine enhances salt tolerance of *Glycyrrhiza uralensis* through multiple pathways. BMC Plant Biol. 2024;24(1):165. doi: 10.1186/s12870-024-04851-w. 38431542 PMC10908008

[11] Archibald JM. Endosymbiosis and eukaryotic cell evolution. Curr Biol. 2015;25(19):R911–R921. doi: 10.1016/j.cub.2015.07.055. 26439354

[12] Zhang T, Li M, Zhu X, Guo M, Shu Y. Comparative chloroplast genomes analysis provided adaptive evolution insights in medicago ruthenica. Int J Mol Sci. 2024;25(16):8689. doi: 10.3390/ijms25168689. 39201375 PMC11354556

[13] Jarvis P, López-Juez E. Biogenesis and homeostasis of chloroplasts and other plastids. Nat Rev Mol Cell Biol. 2013;14(12):787–802. doi: 10.1038/nrm3702. 24263360

[14] Wang X, Chen Z, Sui N. Sensitivity and responses of chloroplasts to salt stress in plants. Front Plant Sci. 2024;15:1374086. doi: 10.3389/fpls.2024.1374086. 38693929 PMC11061501

[15] Wicke S, Schneeweiss GM, dePamphilis CW, Müller KF, Quandt D. The evolution of the plastid chromosome in land plants: gene content, gene order, gene function. Plant Mol Biol. 2011;76(3-5):273–297. doi: 10.1007/s11103-011-9762-4. 21424877 PMC3104136

[16] Yang J, Vázquez L, Chen X, Li H, Zhang H, Liu Z, Zhao G. Development of chloroplast and nuclear DNA markers for chinese oaks (quercus subgenus quercus) and assessment of their utility as dna barcodes. Front Plant Sci. 2017;8:816. doi: 10.3389/fpls.2017.00816. 28579999 PMC5437370

[17] Xiao S, Xu P, Deng Y. Comparative analysis of chloroplast genomes of cultivars and wild species of sweetpotato (*Ipomoea batatas* [L.] Lam). BMC Genomics. 2021;22(1):262. doi: 10.1186/s12864-021-07684-1. 33849443 PMC8042981

[18] Liu H, Zhao W, Hua W, Liu J. A large-scale population based organelle pan-genomes construction and phylogeny analysis reveal the genetic diversity and the evolutionary origins of chloroplast and mitochondrion in *Brassica napus* L. BMC genomics. 2023;24(1):716. doi: 10.1186/s12864-023-09812-5. PMC10680221 38012560

[19] Wu LW, Fan Ph, Cai JY, Zang C, Lin Y, Xu Z, Gao W, Song J, Yao H. Comparative genomics and phylogenomics of the genus *Glycyrrhiza* (Fabaceae) based on chloroplast genomes. Front Pharmacol. 2024;15:1371390. doi: 10.3389/fphar.2024.1371390. 38515836 PMC10955637

[20] Jiang WL, Tan W, Gao H, Yu X, Zhang H, Bian Y, Wang Y, Tian X. Transcriptome and complete chloroplast genome of *Glycyrrhiza inflata* and comparative analyses with the other two licorice species. Genomics. 2020;112(6):4179–4188. doi: 10.1016/j.ygeno.2020.07.007. 32650098

[21] Frazer KA, Pachter L, Poliakov A, Rubin EM, Dubchak I. VISTA: computational tools for comparative genomics. Nucleic Acids Res. 2004;32:W273–W279. doi: 10.1093/nar/gkh458. 15215394 PMC441596

[22] Bolger AM, Lohse M, Usadel B. Trimmomatic: a flexible trimmer for Illumina sequence data. Bioinformatics. 2014;30(15):2114–2120. doi: 10.1093/bioinformatics/btu170. 24695404 PMC4103590

[23] Li H. Aligning sequence reads, clone sequences and assembly contigs with BWA-MEM. arXiv 2013, arXiv:1303.3997.

[24] Li H, Handsaker B, Wysoker A, Fennell T, Ruan J, Homer N, Marth G, Abecasis G, Durbin R. The sequence alignment/map format and SAMtools. Bioinformatics. 2009;25(16):2078–2079. doi: 10.1093/bioinformatics/btp352. 19505943 PMC2723002

[25] McKenna A, Hanna M, Banks E, Sivachenko A, Cibulskis K, Kernytsky A, Garimella K, Altshuler D, Gabriel S, Daly M, et al. The genome analysis toolkit: a MapReduce framework for analyzing next-generation DNA sequencing data. Genome Res. 2010;20(9):1297–1303. doi: 10.1101/gr.107524.110. 20644199 PMC2928508

[26] Katoh K, Misawa K, Kuma K, Miyata T. MAFFT: a novel method for rapid multiple sequence alignment based on fast Fourier transform. Nucleic Acids Res. 2002;30(14):3059–3066. 12136088 10.1093/nar/gkf436 PMC135756

[27] Rozas J, Ferrer-Mata A, Sánchez-DelBarrio JC, Guirao-Rico S, Librado P, Ramos-Onsins SE, Sánchez-Gracia A. DnaSP 6: DNA sequence polymorphism analysis of large data sets. Mol Biol Evol. 2017;34(12):3299–3302. doi: 10.1093/molbev/msx248. 29029172

[28] Zhang Z. KaKs_calculator 3.0: calculating selective pressure on coding and non-coding sequences. Genom Proteom Bioinform. 2022;20(3):536–540. doi: 10.1016/j.gpb.2021.12.002. PMC9801026 34990803

[29] Leigh J, Bryant D, Nakagawa S. POPART: full-feature software for haplotype network construction. Methods Ecol. Evol. 2015;6:1110–1116.

[30] Danecek P, Auton A, Abecasis G, Albers CA, Banks E, DePristo MA, Handsaker RE, Lunter G, Marth GT, Sherry ST, et al. The variant call format and VCFtools. Bioinformatics. 2011;27(15):2156–2158. doi: 10.1093/bioinformatics/btr330. 21653522 PMC3137218

[31] Purcell S, Neale B, Todd-Brown K, Thomas L, Ferreira MA, Bender D, Maller J, Sklar P, de Bakker PI, Daly MJ, et al. PLINK: a tool set for whole-genome association and population-based linkage analyses. Am J Hum Genet. 2007;81(3):559–575. doi: 10.1086/519795. 17701901 PMC1950838

[32] Minh BQ, Schmidt HA, Chernomor O, Schrempf D, Woodhams MD, von Haeseler A, Lanfear R. IQ-TREE 2: new models and efficient methods for phylogenetic inference in the genomic era. Mol Biol Evol. 2020;37(8):2461. doi: 10.1093/molbev/msaa131. 32011700 PMC7182206

[33] Rambaut A. FigTree, a graphical viewer of phylogenetic trees. Institute of Evolutionary Biology University of Edinburgh; 2009. Available online: http://tree.bio.ed.ac.uk/software/figtree.

[34] Alexander DH, Novembre J, Lange K. Fast model-based estimation of ancestry in unrelated individuals. Genome Res. 2009;19(9):1655–1664. doi: 10.1101/gr.094052.109. 19648217 PMC2752134

[35] Patro R, Duggal G, Love MI, Irizarry RA, Kingsford C. Salmon provides fast and bias-aware quantification of transcript expression. Nat Methods. 2017;14(4):417–419. doi: 10.1038/nmeth.4197. 28263959 PMC5600148

[36] Wu S, Liu W, Aljohi HA, Alromaih SA, Alanazi IO, Lin Q, Yu J, Hu S. REDO: RNA editing detection in plant organelles based on variant calling results. J Comput Biol. 2018;25(5):509–516. doi: 10.1089/cmb.2017.0214. 29641228

[37] Zhang T, Chen X, Yan W, Li M, Huang W, Liu Q, Guo C, Shu Y. Comparative analysis of chloroplast pan-genomes and transcriptomics reveals cold adaptation in medicago sativa. Int J Mol Sci. 2024;25(3):1776. doi: 10.3390/ijms25031776. 38339052 PMC10855486

[38] Meng F, Luo Q, Wang Q, Zhang X, Qi Z, Xu F, Lei X, Cao Y, Chow WS, Sun G. Physiological and proteomic responses to salt stress in chloroplasts of diploid and tetraploid black locust (*Robinia pseudoacacia* L). Sci Rep. 2016;6:23098. doi: 10.1038/srep23098. 26975701 PMC4791547

[39] Cai XL, Landis JB, Wang HX, Wang JH, Zhu ZX, Wang HF. Plastome structure and phylogenetic relationships of Styracaceae (Ericales). BMC Ecol Evol. 2021;21(1):103. doi: 10.1186/s12862-021-01827-4. 34049486 PMC8161964

[40] Brancato D, Bruno F, Coniglio E, Sturiale V, Saccone S, Federico C. The chromatin organization close to SNP rs12913832, involved in eye color variation, is evolutionary conserved in vertebrates. Int J Mol Sci. 2024;25(12):6602. doi: 10.3390/ijms25126602. 38928306 PMC11204186

[41] Fu CN, Wu CS, Ye LJ, Mo Z, Liu J, Chang Y, Li D, Chaw S, Gao L. Prevalence of isomeric plastomes and effectiveness of plastome super-barcodes in yews (Taxus) worldwide. Sci Rep. 2019;9(1):2773. doi: 10.1038/s41598-019-39161-x. 30808961 PMC6391452

[42] Lee K. Relocation of chloroplast proteins from cytosols into chloroplasts. Plant Signal Behav. 2023;18(1):2258321. doi: 10.1080/15592324.2023.2258321. 37707988 PMC10503445

[43] Cao H, Han Y, Cheng Z, Lv Q, Pompelli MF, Pereira JD, Araújo WL. Long exposure to salt stress in *Jatropha curcas* leads to stronger damage to the chloroplast ultrastructure and its functionality than the stomatal function. Forests. 2023;14:1868. doi: 10.3390/f14091868.

[44] Pirasteh H, Emam Y. Modulation of oxidative damage due to salt stress using salicylic acid in *Hordeum vulgare*. Arch Agron Soil Sci. 2018;64(9):1268–1277. doi: 10.1080/03650340.2018.1423556.

[45] Wu X, Lin S, Chen N, et al. Study on the molecular evolution of the psaA gene from ferns. Plant Sci J. 2017;35(2):177–185.

[46] Moin M, Saha A, Bakshi A, Madhav MS, Kirti PB. Constitutive expression of ribosomal protein L6 modulates salt tolerance in rice transgenic plants. Gene. 2021;789:145670. doi: 10.1016/j.gene.2021.145670. 33892070

[47] Lan Y, Chen Q, Kong M, Liu Y, Lyu MA, Perveen S, Mi H. PetM Is essential for the stabilization and function of the cytochrome b6f complex in arabidopsis. Plant Cell Physiol. 2021;62(10):1603–1614. doi: 10.1093/pcp/pcab116. 34283246

[48] Mohammed T, Firoz A, Ramadan AM. RNA editing in chloroplast: advancements and opportunities. Curr Issues Mol Biol. 2022;44(11):5593–5604. doi: 10.3390/cimb44110379. 36421663 PMC9688838

[49] Ciuzan O, Hancock J, Pamfil D, Wilson I, Ladomery M. The evolutionarily conserved multifunctional glycine-rich RNA-binding proteins play key roles in development and stress adaptation. Physiol Plant. 2015;153(1):1–11. doi: 10.1111/ppl.12286. 25243592

[50] Chotewutmontri P, Barkan A. Light-induced psbA translation in plants is triggered by photosystem II damage via an assembly-linked autoregulatory circuit. Proc Natl Acad Sci U S A. 2020;117(35):21775–21784. doi: 10.1073/pnas.2007833117. 32817480 PMC7474643

