# Supplementary material for: Comparative analysis of chloroplast genomes and transcriptomics reveals the adaptation of Glycyrrhiza to salt stress
Source: Plant Signal Behav. 2025 Nov 13;20(1):2584568. doi: 10.1080/15592324.2025.2584568 (PMC12622338; doi:10.1080/15592324.2025.2584568)
Supplement: Supplementary material — Table S4 Statistics on RNA editing sites in Glycyrrhiza. [file KPSB_A_2584568_SM2296.docx]

**Table S3** Statistical analysis of haplotype numbers in sixty *Glycyrrhiza uralensi*

| Haplotype | S | U | Y |
| --- | --- | --- | --- |
| Hap_1 | 1 | 0 | 0 |
| Hap_2 | 1 | 0 | 0 |
| Hap_3 | 1 | 0 | 0 |
| Hap_4 | 1 | 0 | 0 |
| Hap_5 | 0 | 0 | 1 |
| Hap_6 | 0 | 0 | 1 |
| Hap_7 | 0 | 0 | 1 |
| Hap_8 | 0 | 0 | 1 |
| Hap_9 | 0 | 0 | 1 |
| Hap_10 | 0 | 0 | 1 |
| Hap_11 | 0 | 0 | 1 |
| Hap_12 | 0 | 0 | 1 |
| Hap_13 | 0 | 0 | 1 |
| Hap_14 | 0 | 0 | 1 |
| Hap_15 | 1 | 0 | 1 |
| Hap_16 | 0 | 0 | 1 |
| Hap_17 | 0 | 0 | 1 |
| Hap_18 | 0 | 0 | 1 |
| Hap_19 | 0 | 0 | 1 |
| Hap_20 | 0 | 0 | 1 |
| Hap_21 | 0 | 0 | 1 |
| Hap_22 | 0 | 0 | 1 |
| Hap_23 | 0 | 0 | 2 |
| Hap_24 | 0 | 0 | 1 |
| Hap_25 | 1 | 0 | 0 |
| Hap_26 | 0 | 1 | 0 |
| Hap_27 | 2 | 1 | 0 |
| Hap_28 | 0 | 1 | 0 |
| Hap_29 | 0 | 1 | 0 |
| Hap_30 | 0 | 1 | 0 |
| Hap_31 | 0 | 1 | 0 |
| Hap_32 | 0 | 1 | 0 |
| Hap_33 | 0 | 1 | 0 |
| Hap_34 | 0 | 1 | 0 |
| Hap_35 | 0 | 1 | 0 |
| Hap_36 | 0 | 1 | 0 |
| Hap_37 | 0 | 1 | 0 |
| Hap_38 | 0 | 1 | 0 |
| Hap_39 | 0 | 1 | 0 |
| Hap_40 | 0 | 1 | 0 |
| Hap_41 | 0 | 1 | 0 |
| Hap_42 | 0 | 1 | 0 |
| Hap_43 | 0 | 1 | 0 |
| Hap_44 | 0 | 1 | 0 |
| Hap_45 | 0 | 1 | 0 |
| Hap_46 | 0 | 1 | 0 |
| Hap_47 | 1 | 0 | 0 |
| Hap_48 | 1 | 0 | 0 |
| Hap_49 | 1 | 0 | 0 |
| Hap_50 | 1 | 0 | 0 |
| Hap_51 | 1 | 0 | 0 |
| Hap_52 | 1 | 0 | 0 |
| Hap_53 | 1 | 0 | 0 |
| Hap_54 | 1 | 0 | 0 |
| Hap_55 | 1 | 0 | 0 |
| Hap_56 | 2 | 0 | 0 |
| Hap_57 | 1 | 0 | 0 |
