# Supplementary material for: Comparative analysis of chloroplast genomes and transcriptomics reveals the adaptation of Glycyrrhiza to salt stress
Source: Plant Signal Behav. 2025 Nov 13;20(1):2584568. doi: 10.1080/15592324.2025.2584568 (PMC12622338; doi:10.1080/15592324.2025.2584568)
Supplement: Supplementary material — Table S3 Statistical analysis of haplotype numbers in sixty Glycyrrhiza uralensi. [file KPSB_A_2584568_SM2297.docx]

**Table S4** Statistics on RNA editing sites in *Glycyrrhiza*.

| Gene | 5379 | 5383 | 5385 | 5386 | 5391 | 5392 | 5395 | 5398 | 5400 | 5401 |
| --- | --- | --- | --- | --- | --- | --- | --- | --- | --- | --- |
| ndhJ | 1 | 0 | 0 | 0 | 0 | 0 | 0 | 1 | 0 | 0 |
| psaA | 1 | 0 | 0 | 0 | 0 | 0 | 0 | 0 | 0 | 0 |
| psaB | 2 | 1 | 2 | 1 | 2 | 1 | 0 | 1 | 0 | 2 |
| rpoB | 1 | 1 | 3 | 5 | 5 | 1 | 0 | 2 | 0 | 0 |
| rpoA | 1 | 0 | 0 | 1 | 0 | 0 | 0 | 1 | 0 | 1 |
| rpl2 | 2 | 2 | 0 | 2 | 1 | 2 | 0 | 2 | 0 | 1 |
| ycf2 | 2 | 2 | 0 | 2 | 0 | 5 | 0 | 0 | 0 | 5 |
| rrn16 | 4 | 3 | 2 | 4 | 4 | 3 | 4 | 0 | 4 | 4 |
| rrn23 | 4 | 3 | 3 | 5 | 4 | 11 | 4 | 2 | 3 | 4 |
| psbD | 0 | 2 | 0 | 1 | 0 | 4 | 2 | 0 | 0 | 2 |
| psbN | 0 | 4 | 5 | 1 | 0 | 1 | 0 | 2 | 4 | 1 |
| cemA | 0 | 0 | 3 | 0 | 0 | 0 | 1 | 0 | 9 | 0 |
| psbB | 0 | 0 | 1 | 0 | 0 | 0 | 0 | 0 | 0 | 0 |
| rpl14 | 0 | 0 | 1 | 0 | 0 | 0 | 0 | 0 | 1 | 0 |
| atpI | 0 | 0 | 0 | 2 | 0 | 0 | 0 | 3 | 0 | 0 |
| rbcL | 0 | 0 | 0 | 0 | 1 | 7 | 0 | 0 | 0 | 0 |
| psbA | 0 | 0 | 0 | 0 | 0 | 24 | 0 | 0 | 4 | 0 |
| atpA | 0 | 0 | 0 | 0 | 0 | 2 | 0 | 0 | 0 | 0 |
| psbT | 0 | 0 | 0 | 0 | 0 | 1 | 0 | 0 | 0 | 0 |
