# Supplementary material for: Comparative analysis of chloroplast genomes and transcriptomics reveals the adaptation of Glycyrrhiza to salt stress
Source: Plant Signal Behav. 2025 Nov 13;20(1):2584568. doi: 10.1080/15592324.2025.2584568 (PMC12622338; doi:10.1080/15592324.2025.2584568)
Supplement: Supplementary material — Table S2 Information of sixty Glycyrrhiza uralensis. [file KPSB_A_2584568_SM2298.docx]

**Table S2** Information of sixty *Glycyrrhiza uralensis*

| Run | Location | Name | Species |
| --- | --- | --- | --- |
| SRR14570085 | Central region | SW-10 | Glycyrrhiza uralensis |
| SRR14570086 | Central region | SW-09 | Glycyrrhiza uralensis |
| SRR14570087 | Central region | SW-08 | Glycyrrhiza uralensis |
| SRR14570088 | Central region | SW-07 | Glycyrrhiza uralensis |
| SRR14570089 | Southern region | YW-20 | Glycyrrhiza uralensis |
| SRR14570090 | Southern region | YW-19 | Glycyrrhiza uralensis |
| SRR14570091 | Southern region | YW-18 | Glycyrrhiza uralensis |
| SRR14570092 | Southern region | YW-17 | Glycyrrhiza uralensis |
| SRR14570093 | Southern region | YW-16 | Glycyrrhiza uralensis |
| SRR14570094 | Southern region | YW-15 | Glycyrrhiza uralensis |
| SRR14570095 | Southern region | YW-14 | Glycyrrhiza uralensis |
| SRR14570096 | Southern region | YW-13 | Glycyrrhiza uralensis |
| SRR14570097 | Southern region | YW-12 | Glycyrrhiza uralensis |
| SRR14570098 | Southern region | YW-11 | Glycyrrhiza uralensis |
| SRR14570099 | Central region | SW-06 | Glycyrrhiza uralensis |
| SRR14570100 | Southern region | YW-10 | Glycyrrhiza uralensis |
| SRR14570101 | Southern region | YW-09 | Glycyrrhiza uralensis |
| SRR14570102 | Southern region | YW-08 | Glycyrrhiza uralensis |
| SRR14570103 | Southern region | YW-07 | Glycyrrhiza uralensis |
| SRR14570104 | Southern region | YW-06 | Glycyrrhiza uralensis |
| SRR14570105 | Southern region | YW-05 | Glycyrrhiza uralensis |
| SRR14570106 | Southern region | YW-04 | Glycyrrhiza uralensis |
| SRR14570107 | Southern region | YW-03 | Glycyrrhiza uralensis |
| SRR14570108 | Southern region | YW-02 | Glycyrrhiza uralensis |
| SRR14570109 | Southern region | YW-01 | Glycyrrhiza uralensis |
| SRR14570110 | Central region | SW-5 | Glycyrrhiza uralensis |
| SRR14570111 | Eastern region | UW-20 | Glycyrrhiza uralensis |
| SRR14570112 | Eastern region | UW-19 | Glycyrrhiza uralensis |
| SRR14570113 | Eastern region | UW-18 | Glycyrrhiza uralensis |
| SRR14570114 | Eastern region | UW-17 | Glycyrrhiza uralensis |
| SRR14570115 | Eastern region | UW-16 | Glycyrrhiza uralensis |
| SRR14570116 | Eastern region | UW-15 | Glycyrrhiza uralensis |
| SRR14570117 | Eastern region | UW-14 | Glycyrrhiza uralensis |
| SRR14570118 | Eastern region | UW-13 | Glycyrrhiza uralensis |
| SRR14570119 | Eastern region | UW-12 | Glycyrrhiza uralensis |
| SRR14570120 | Eastern region | UW-11 | Glycyrrhiza uralensis |
| SRR14570121 | Eastern region | SW-04 | Glycyrrhiza uralensis |
| SRR14570122 | Eastern region | UW-10 | Glycyrrhiza uralensis |
| SRR14570123 | Eastern region | UW-09 | Glycyrrhiza uralensis |
| SRR14570124 | Eastern region | UW-08 | Glycyrrhiza uralensis |
| SRR14570125 | Eastern region | UW-07 | Glycyrrhiza uralensis |
| SRR14570126 | Eastern region | UW-06 | Glycyrrhiza uralensis |
| SRR14570127 | Eastern region | UW-05 | Glycyrrhiza uralensis |
| SRR14570128 | Eastern region | UW-04 | Glycyrrhiza uralensis |
| SRR14570129 | Eastern region | UW-03 | Glycyrrhiza uralensis |
| SRR14570130 | Eastern region | UW-02 | Glycyrrhiza uralensis |
| SRR14570131 | Eastern region | UW-01 | Glycyrrhiza uralensis |
| SRR14570132 | Central region | SW-03 | Glycyrrhiza uralensis |
| SRR14570133 | Central region | SW-20 | Glycyrrhiza uralensis |
| SRR14570134 | Central region | SW-19 | Glycyrrhiza uralensis |
| SRR14570135 | Central region | SW-18 | Glycyrrhiza uralensis |
| SRR14570136 | Central region | SW-17 | Glycyrrhiza uralensis |
| SRR14570137 | Central region | SW-16 | Glycyrrhiza uralensis |
| SRR14570138 | Central region | SW-15 | Glycyrrhiza uralensis |
| SRR14570139 | Central region | SW-14 | Glycyrrhiza uralensis |
| SRR14570140 | Central region | SW-13 | Glycyrrhiza uralensis |
| SRR14570141 | Central region | SW-12 | Glycyrrhiza uralensis |
| SRR14570142 | Central region | SW-11 | Glycyrrhiza uralensis |
| SRR14570143 | Central region | SW-02 | Glycyrrhiza uralensis |
| SRR14570144 | Central region | SW-01 | Glycyrrhiza uralensis |
