# Supplementary material for: Comparative analysis of chloroplast genomes and transcriptomics reveals the adaptation of Glycyrrhiza to salt stress
Source: Plant Signal Behav. 2025 Nov 13;20(1):2584568. doi: 10.1080/15592324.2025.2584568 (PMC12622338; doi:10.1080/15592324.2025.2584568)
Supplement: Supplementary material — Table S1 Information of seven Glycyrrhiza varieties. [file KPSB_A_2584568_SM2299.docx]

**Table S1** Information of senven *Glycyrrhiza* varieties

| Run | Location | Name | Species |
| --- | --- | --- | --- |
| MH321931 | China | Y1 | Glycyrrhiza inflata |
| MN562092 | China | Y2 | Glycyrrhiza inflata |
| KU862308 | China | Y3 | Glycyrrhiza uralensis |
| KY038482 | China | Y4 | Glycyrrhiza lepidota |
| PP119340 | China | Y5 | Glycyrrhiza inflata |
| PP119343 | China | Y6 | Glycyrrhiza squamulosa |
| MN199032 | China | Y7 | Glycyrrhiza uralensis |
